# Supplementary material for: Latrophilin-2 mediates fluid shear stress mechanotransduction at endothelial junctions
Source: EMBO J. 2024 Jun 17;43(15):3175–91. doi: 10.1038/s44318-024-00142-0 (PMC11294477; doi:10.1038/s44318-024-00142-0)
Supplement: Supplementary file 1 — Appendix [file 44318_2024_142_MOESM1_ESM.pdf]

Appendix

Table of contents

Appendix Figure S1 (Gαi and Gαq/11 in flow) …………………………………….. p.2-3

Appendix Figure S2 (Confirmation of knockdown and rescues of Gα proteins)……. p.4

Appendix Figure S3 (Pulldown assay for Gα activation)…………………………… p.5-6

Appendix Figure S4 (Proteomic identification of Latrophilin) ……………………... p.7-8

Appendix Figure S5 (Latrophilin expression and contribution to cell orientation)... p.9-10

Appendix Figure S6 (Subcellular localization of Latrophilin-2)…………………….. p.11

Appendix Figure S7 (Latrophilin functions under ligand stimulation)……………… p.12

Appendix Figure S8 (Validation of knockdown and rescues by Latrophilin mutants).. p.13

Appendix Figure S9 (Validation of latrophilin CRISPR mutant zebrafish)…….……. p.14

Appendix Figure S10
(SNP analysis of human Adgrl2 gene locus and links to cardiovascular disease)……………………………………………………………………………. p.15

Appendix Figure S11 (Latrophilin-mediated endothelial flow signaling pathways and their physiological roles)…………..…………………………………………………… p.16

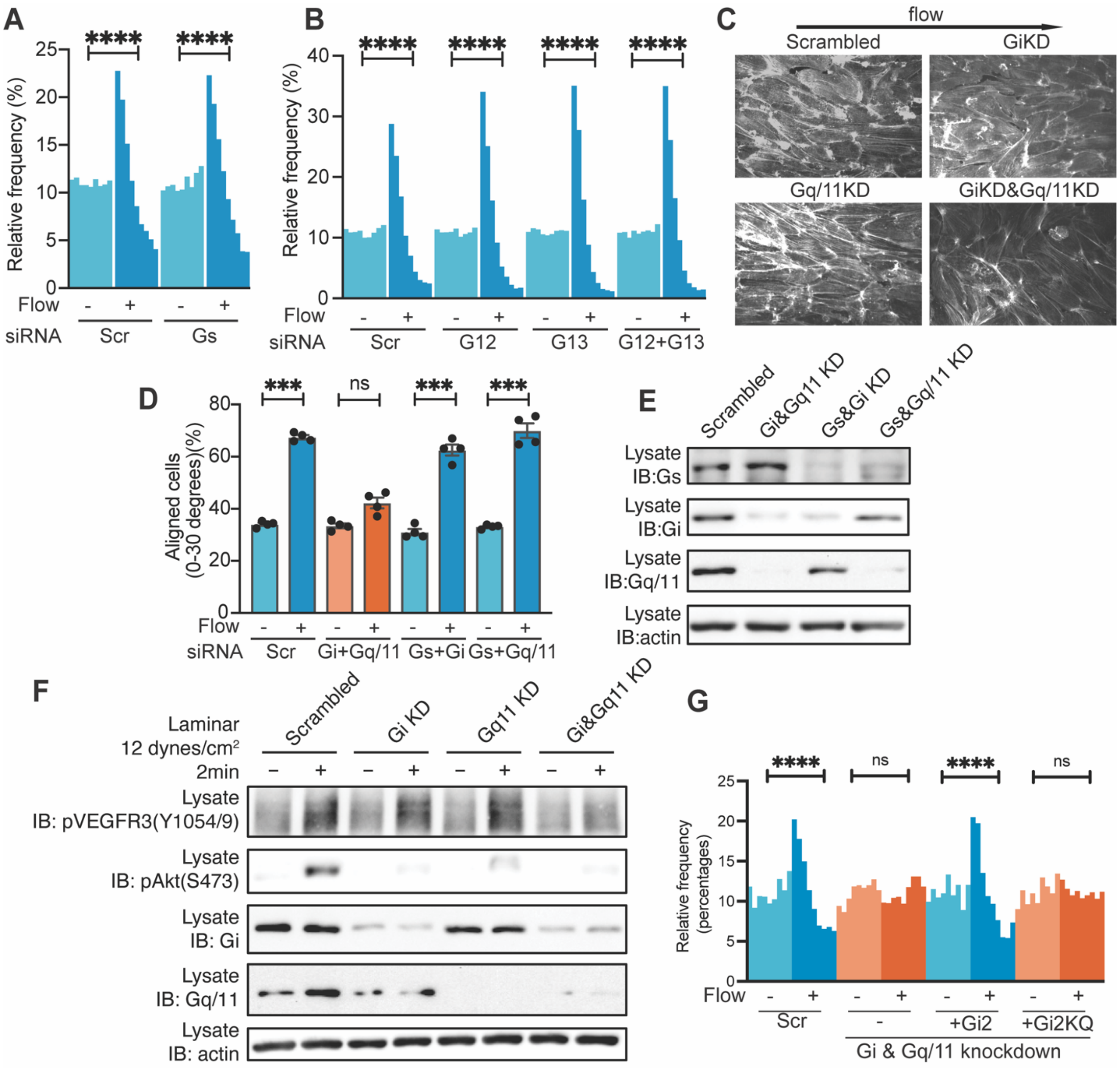
A
B
C
D
E
F
G
Relative frequency (%)
Flow
siRNA
Scr
Gs
G12
G13
G12+G13
flow
Scrambled
GiKD
Gq/11KD
GiKD&Gq/11KD
Aligned cells (0-30 degrees)(%)
ns
Gi+Gq/11
Gs+Gi
Gs+Gq/11
Gi&Gq11 KD
Gs&Gi KD
Gs&Gq/11 KD
Lysate IB:Gs
Lysate IB:Gi
Lysate IB:Gq/11
Lysate IB:actin
Laminar 12 dynes/cm2 2min
Gi KD
Gq11 KD
Lysate IB: pVEGFR3(Y1054/9)
Lysate IB: pAkt(S473)
Lysate IB: Gi
Lysate IB: Gq/11
Lysate IB: actin
Relative frequency (percentages)
+Gi2
+Gi2KQ
Gi & Gq/11 knockdown

<u>Appendix Figure S1. Gαi and Gαq/11 in flow.</u>

A. HUVECs after Gαs or control knockdown were subjected to FSS at 12 dynes/cm^2 for 16 hours and cell alignment quantified as in Fig 1. ****: $p<0.0001$, one-way ANOVA with Tukey's multiple comparisons test. **B**, HUVECs after Gα12 or/and Gα13 or control knockdown were subjected to FSS and alignment determined as in **A**. ****: $p<0.0001$; one-way ANOVA with Tukey's multiple comparisons test. **C**, Phalloidin staining of cells from Fig. 1**A**. **D**, HUVECs transfected with indicated siRNAs were subjected to FSS and alignment determined as in **A**. ***: $p<0.001$; one-way ANOVA with Tukey's multiple comparisons test. **E**. Confirmation of knockdowns. **F**. Flow-induced activation of VEGFR3 and Akt kinase after knockdown of the indicated Gα proteins. **G**. Rescue of Gq/11 and Gi knockdown by re-expression of siRNA-resistant versions of the wild-type or K307Q Gi2 protein. ****: $p<0.0001$; one-way ANOVA with Tukey's multiple comparison test.

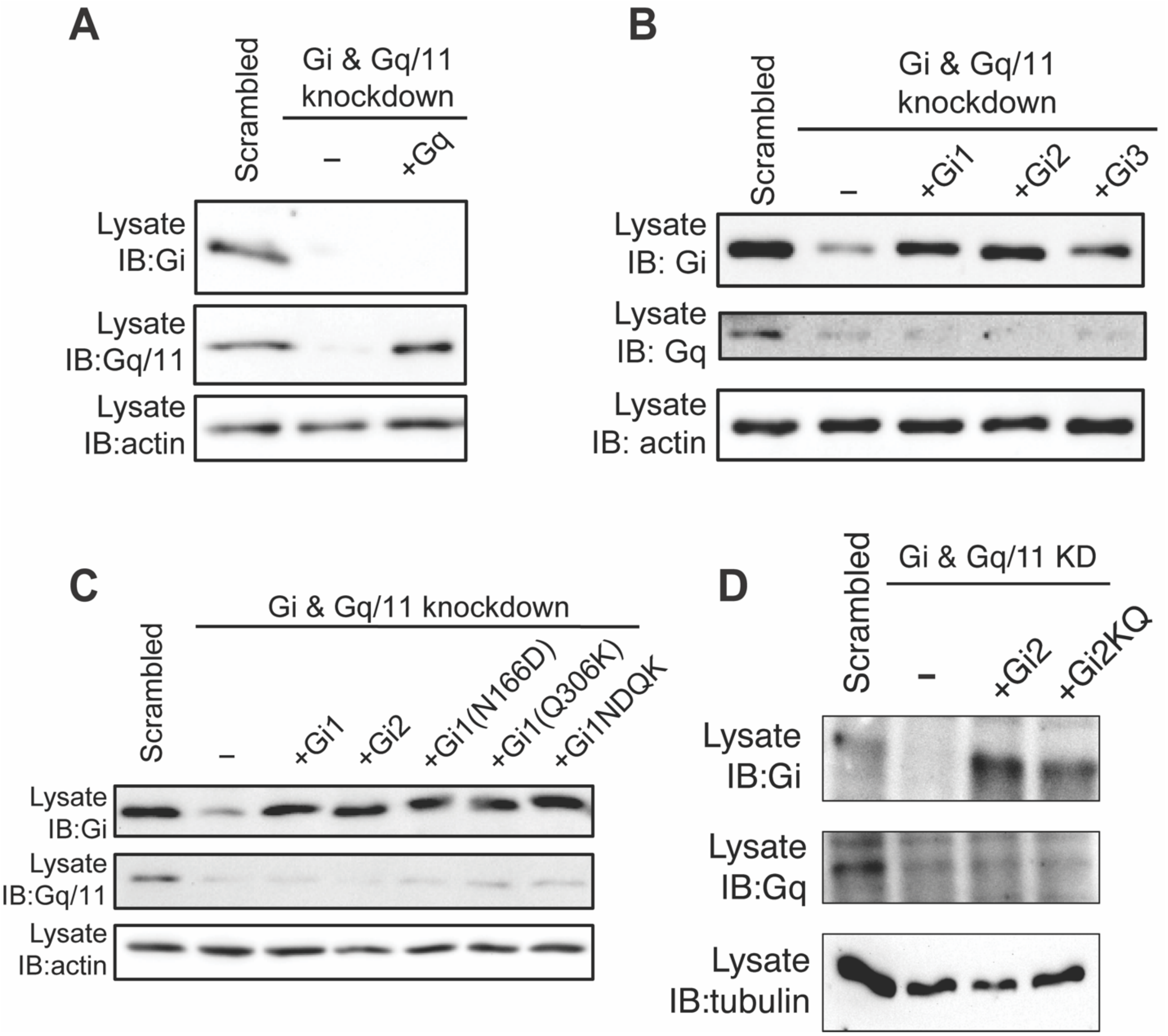

<u>Appendix Figure S2. Confirmation of knockdown and rescues of Gα proteins.</u>

A & B. Confirming knockdowns and rescue for Fig. 1. **D**. **C**. Knockdowns and rescues for Fig. 1**F**.

D. Confirmation of knockdowns and rescue for Appendix fig. S1G

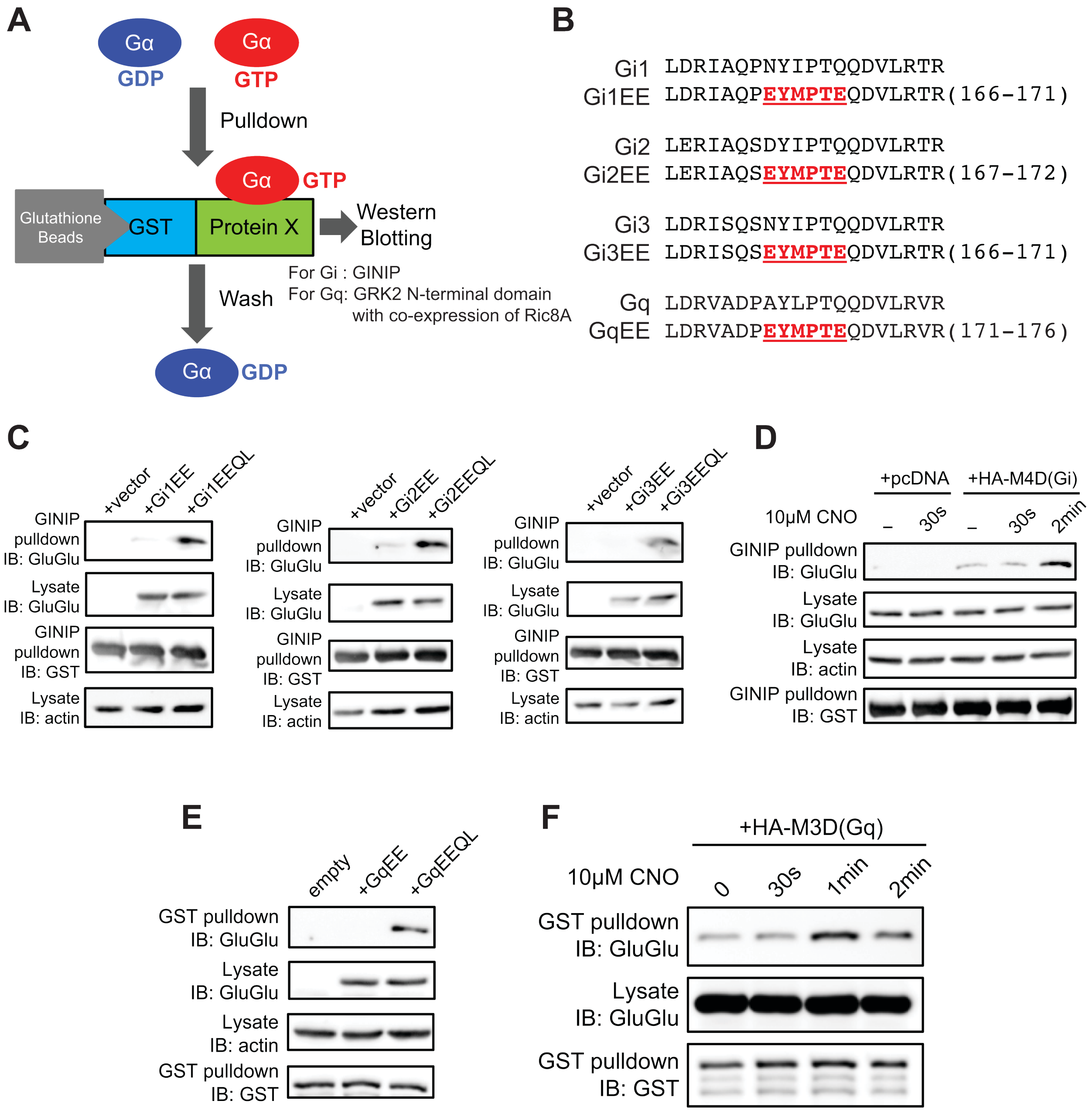
A
Gα
GDP
Gα
GTP
Pulldown
Gα
GTP
Glutathione Beads
GST
Protein X
Western Blotting
Wash
For Gi : GINIP
For Gq: GRK2 N-terminal domain with co-expression of Ric8A
Gα
GDP
B
Gi1 LDRIAQPNYIPTQQDVLRTR
Gi1EE LDRIAQPEYMPTEQDVLRTR(166-171)
Gi2 LERIAQSDYIPTQQDVLRTR
Gi2EE LERIAQSEYMPTEQDVLRTR(167-172)
Gi3 LDRISQSNYIPTQQDVLRTR
Gi3EE LDRISQSEYMPTEQDVLRTR(166-171)
Gq LDRVADPAYLPTQQDVLRVR
GqEE LDRVADPEYMPTEQDVLRVR(171-176)
C
+vector
+Gi1EE
+Gi1EEQL
+Gi2EE
+Gi2EEQL
+Gi3EE
+Gi3EEQL
GINIP pulldown IB: GluGlu
Lysate IB: GluGlu
GINIP pulldown IB: GST
Lysate IB: actin
D
+pcDNA
+HA-M4D(Gi)
10μM CNO
–
30s
2min
GINIP pulldown IB: GluGlu
Lysate IB: GluGlu
Lysate IB: actin
GINIP pulldown IB: GST
E
empty
+GqEE
+GqEEQL
GST pulldown IB: GluGlu
Lysate IB: GluGlu
Lysate IB: actin
GST pulldown IB: GST
F
+HA-M3D(Gq)
10μM CNO
0
30s
1min
2min
GST pulldown IB: GluGlu
Lysate IB: GluGlu
GST pulldown IB: GST

Appendix Figure S3. Pulldown assay for Gα activation

A. Schematic for Gα pulldown assay. As described in Methods, ECs expressing Gα proteins with inserted EE tags and the indicated mutations were treated as indicated; detergent extracts were incubated with control or effector (GINIP or GRK2) beads and bound Gα proteins analyzed by Western blotting. **B**. Amino acid sequences showing the inserted GluGlu tag. **C**. GINIP pulldown assay with WT and constitutively active (QL) Gi. **D**. GINIP pulldown assay for Gi activation by artificial DREADD M4D (Gi-coupling) by its ligand 10μM CNO. N=3. **E**. GRK2N pulldown assay with WT and constitutively active (QL) Gq. **F**. GRK2 pulldown assay with artificial DREADD M3D (Gq-coupling) after addition of 10μM CNO. N=4.

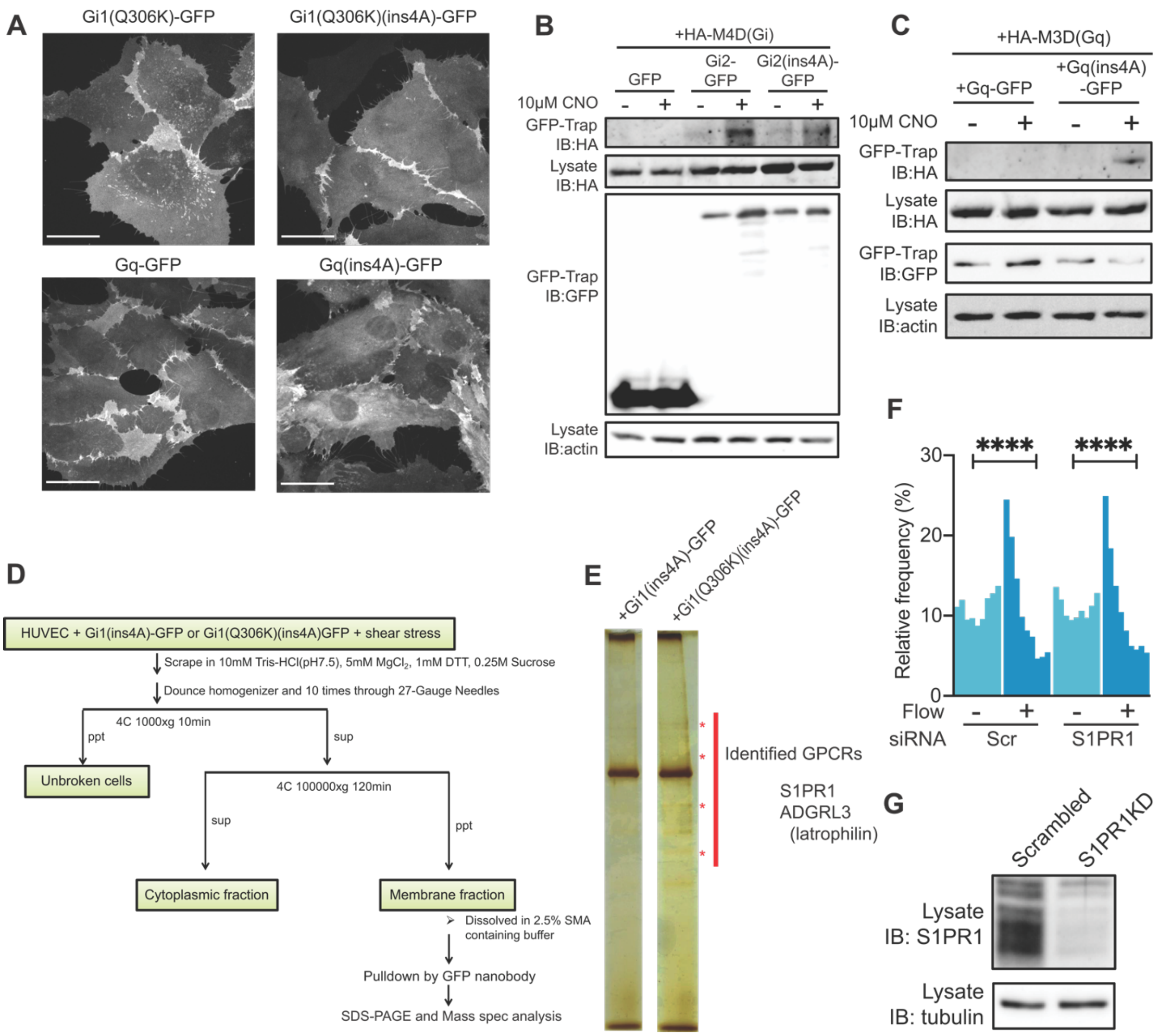
A
Gi1(Q306K)-GFP
Gi1(Q306K)(ins4A)-GFP
Gq-GFP
Gq(ins4A)-GFP
B
+HA-M4D(Gi)
GFP
Gi2-GFP
Gi2(ins4A)-GFP
10μM CNO
GFP-Trap IB:HA
Lysate IB:HA
GFP-Trap IB:GFP
Lysate IB:actin
C
+HA-M3D(Gq)
+Gq-GFP
+Gq(ins4A)-GFP
10μM CNO
GFP-Trap IB:HA
Lysate IB:HA
GFP-Trap IB:GFP
Lysate IB:actin
D
HUVEC + Gi1(ins4A)-GFP or Gi1(Q306K)(ins4A)GFP + shear stress
Scrape in 10mM Tris-HCl(pH7.5), 5mM MgCl2, 1mM DTT, 0.25M Sucrose
Dounce homogenizer and 10 times through 27-Gauge Needles
4C 1000xg 10min
ppt
sup
Unbroken cells
4C 100000xg 120min
sup
ppt
Cytoplasmic fraction
Membrane fraction
Dissolved in 2.5% SMA containing buffer
Pulldown by GFP nanobody
SDS-PAGE and Mass spec analysis
E
+Gi1(ins4A)-GFP
+Gi1(Q306K)(ins4A)-GFP
Identified GPCRs
S1PR1
ADGRL3
(latrophilin)
F
Relative frequency (%)
30
20
10
0
****
****
Flow
siRNA
Scr
S1PR1
G
Scrambled
S1PR1KD
Lysate IB: S1PR1
Lysate IB: tubulin

Appendix Figure S4. Proteomic identification of Latrophilin

A. Subcellular localization of the indicated GFP-labeled Gα proteins. HUVECs expressing the indicated GFP-labeled Gα proteins were imaged using a 60x objective on a Perkin Elmer spinning disk confocal microscope. Scale bar: 30µm. **B**. HEK293T cells expressing Gi2(ins4A)-GFP or its ins4A mutant plus HA-tagged M4D(Gi) were treated with 10 µM CNO or DMSO. Purified membrane fractions were solubilized with SMA polymer and immunoprecipitated with GFP-Trap® beads. Immunoprecipitates were Western blotted for the indicated proteins. **C**. HEK293T cells expressing Gq-GFP or Gq(ins4A)-GFP plus HA-M3D(Gq-coupling) were treated as in **B**. **D**. Workflow for proteomic identification of flow-responsive GPCRs. **E**. Silver staining of pulldowns from HUVECs expressing Gi1(ins4A)-GFP (negative control) or Gi1(Q306K)(ins4A)-GFP (samples). Red asterisks indicate bands specific to Gi1(Q306K)(ins4A)-GFP and identified as GPCRs. **F**, HUVECs transfected with siRNAs targeting S1PR1 were subjected to FSS at 12 dynes/cm^2 for 16 hours and cell alignment quantified as in Fig 1. ****: $p<0.0001$. **G**. S1PR1 knockdown confirmed by Western blotting.

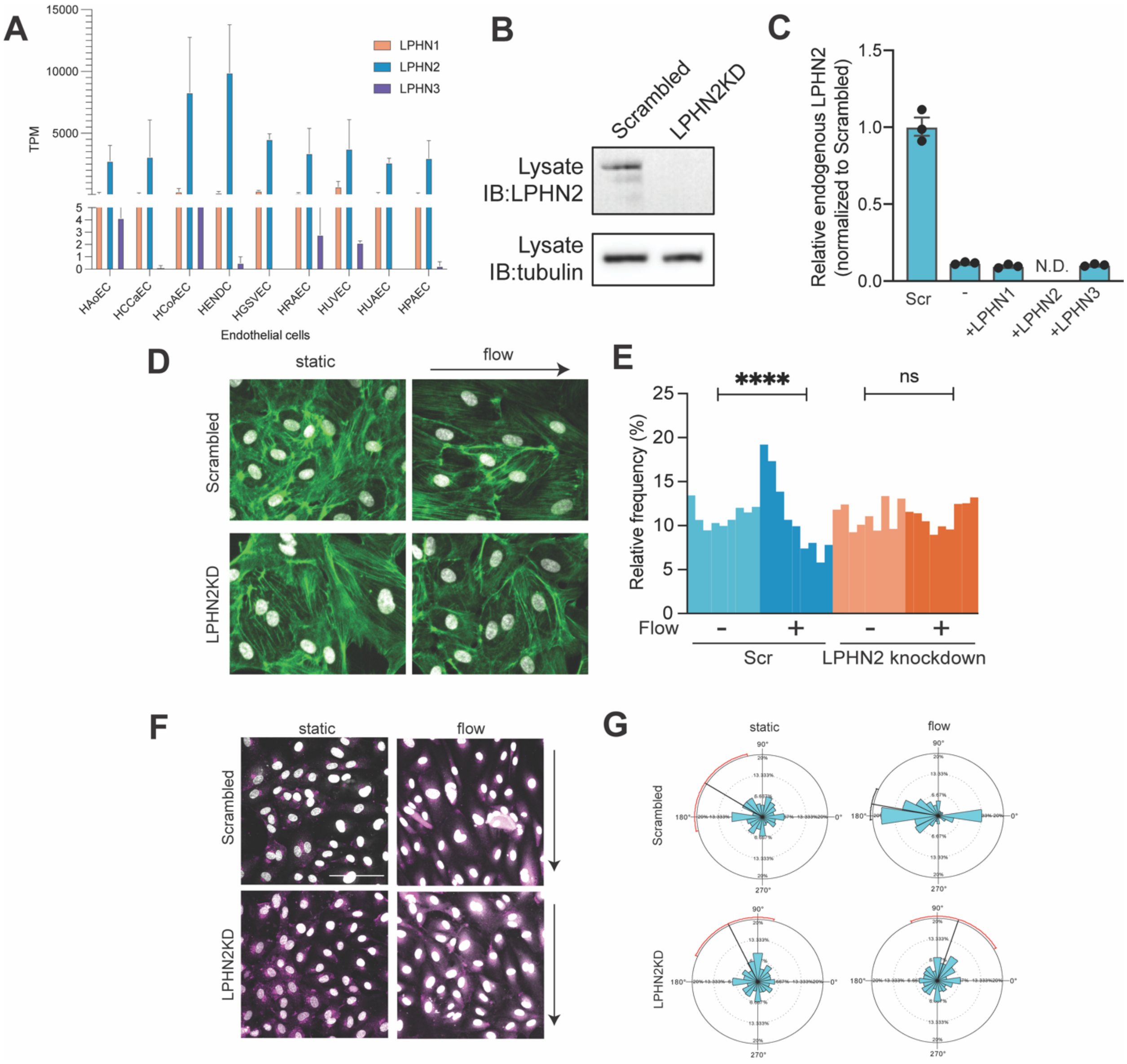
A
15000
10000
5000
TPM
5
4
3
2
1
0
LPHN1
LPHN2
LPHN3
HAoEC
HCCaEC
HCoAEC
HENDC
HGSVEC
HRAEC
HUVEC
HUAEC
HPAEC
Endothelial cells
B
Scrambled
LPHN2KD
Lysate
IB:LPHN2
Lysate
IB:tubulin
C
Relative endogenous LPHN2
(normalized to Scrambled)
1.5
1.0
0.5
0.0
Scr
-
+LPHN1
+LPHN2
+LPHN3
N.D.
D
static
flow
Scrambled
LPHN2KD
E
****
ns
Relative frequency (%)
25
20
15
10
5
0
Flow
-
+
-
+
Scr
LPHN2 knockdown
F
static
flow
Scrambled
LPHN2KD
G
static
flow
Scrambled
LPHN2KD
90°
180°
0°
270°

<u>Appendix Figure S5. Latrophilin expression and contribution to cell orientation</u>

A. Expression of latrophilin isoforms in different endothelial cells obtained from GSE131681 (https://rnakato.github.io/HumanEndothelialEpigenome/). HAoEC: Human aortic endothelial cells, HCCaEC: Human common carotid artery endothelial cells, HCoAEC: Human coronary artery endothelial cells, HENDC: Human endocardial cells, HGSVEC: Human great saphenous vein endothelial cells, HRAEC: Human renal artery endothelial cells, HUVEC: Human umbilical vein endothelial cells, HUAEC: Human umbilical artery endothelial cells, HPAEC: Human pulmonary artery endothelial cells. **B**. Protein level of endogenous LPHN2 following transfection of LPHN2 siRNA. **C**. mRNA levels for LPHN isoforms in Figures 3**B** and 3**H-J**. **D & E**. Human aortic endothelial cells (HAECs) depleted for LPHN2 were subjected to FSS at 12 dynes/cm2 for 16 hours. Cell alignment was quantified as in Fig. 1**A**. ****: $p<0.0001$; one-way ANOVA with Tukey's multiple comparisons test. n=3. **F**. Quantification of Golgi orientation with or without latrophilins under flow by staining of GM130 and Hoechst. Scale bar: 100μm. **G**. Rose plot of the angle of vector from the nucleus center mass to the Golgi center mass. 0° is defined as against the flow direction.

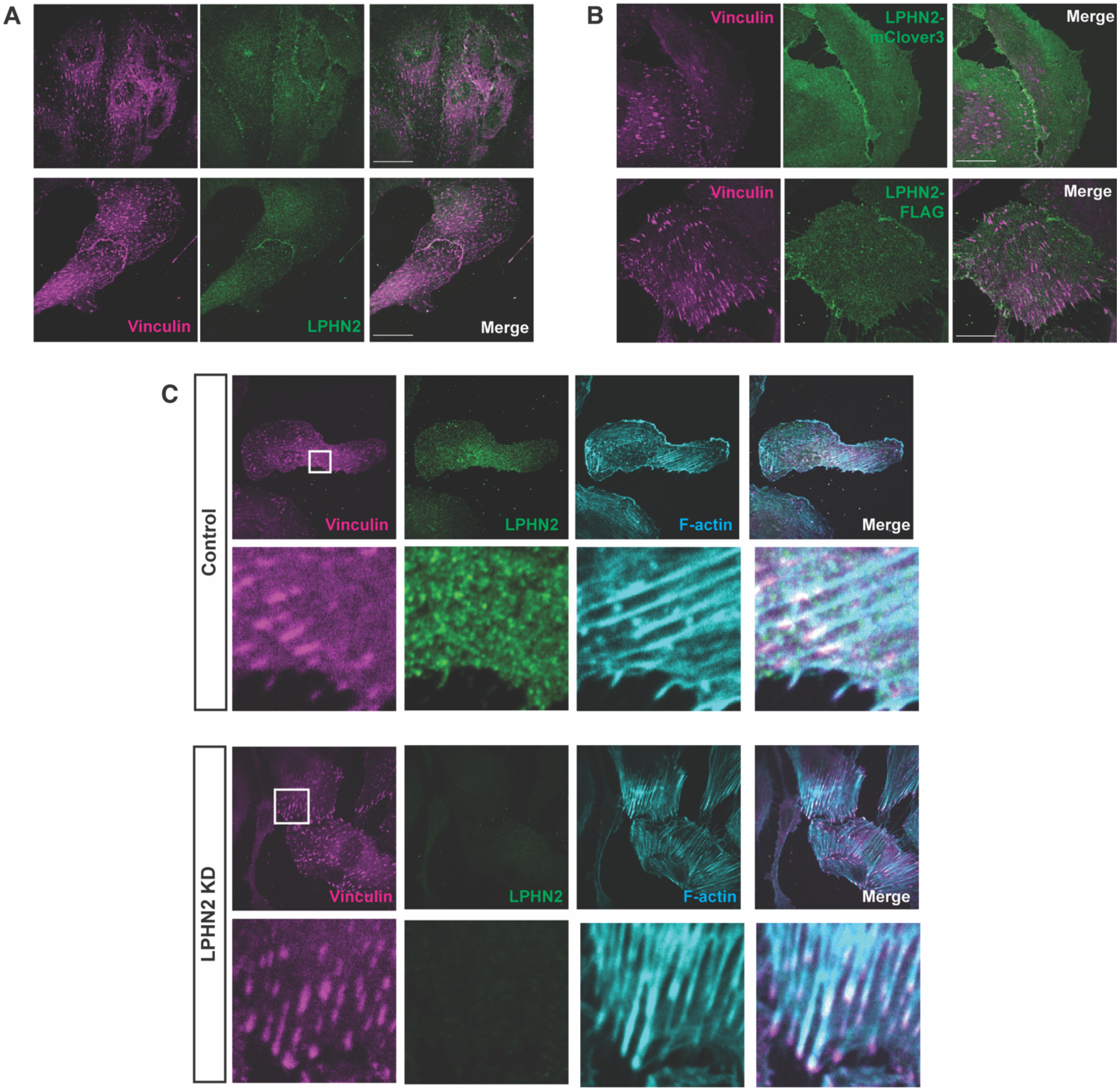

Appendix Figure S6. Subcellular localization of Latrophilin-2

A. Localization of endogenous LPHN2 and the focal adhesion marker vinculin in HUVECs. Scale bar: 20µm. **B**. Localization of overexpressed LPHN2-mClover3 and LPHN2-FLAG in HUVECs. Scale bar: 20µm. **C**. Localization of endogenous LPHN2, vinculin and F-actin in HUVECs with and without knockdown of LPHN2.

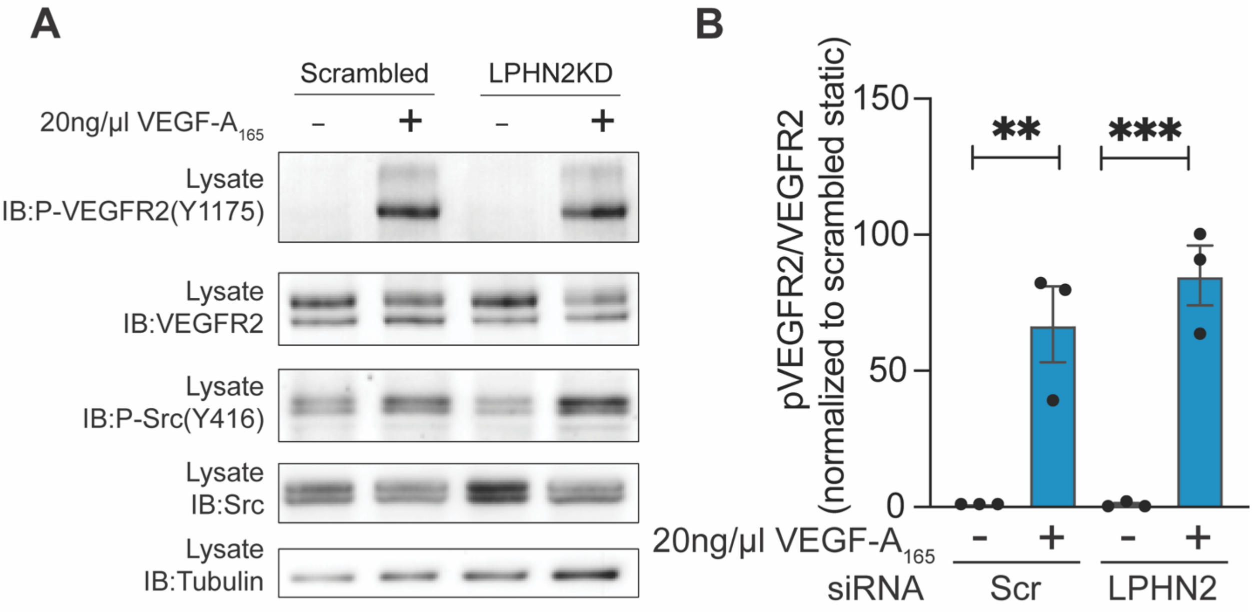

Appendix Figure S7. Latrophilin functions under ligand stimulation

A. VEGF-A induced VEGFR2 phosphorylation ± Latrophilin-2 knockdown. Quantification in **B**.

**: $p=0.0034$, ***: $p=0.0007$; one-way ANOVA with Tukey's multiple comparison test.

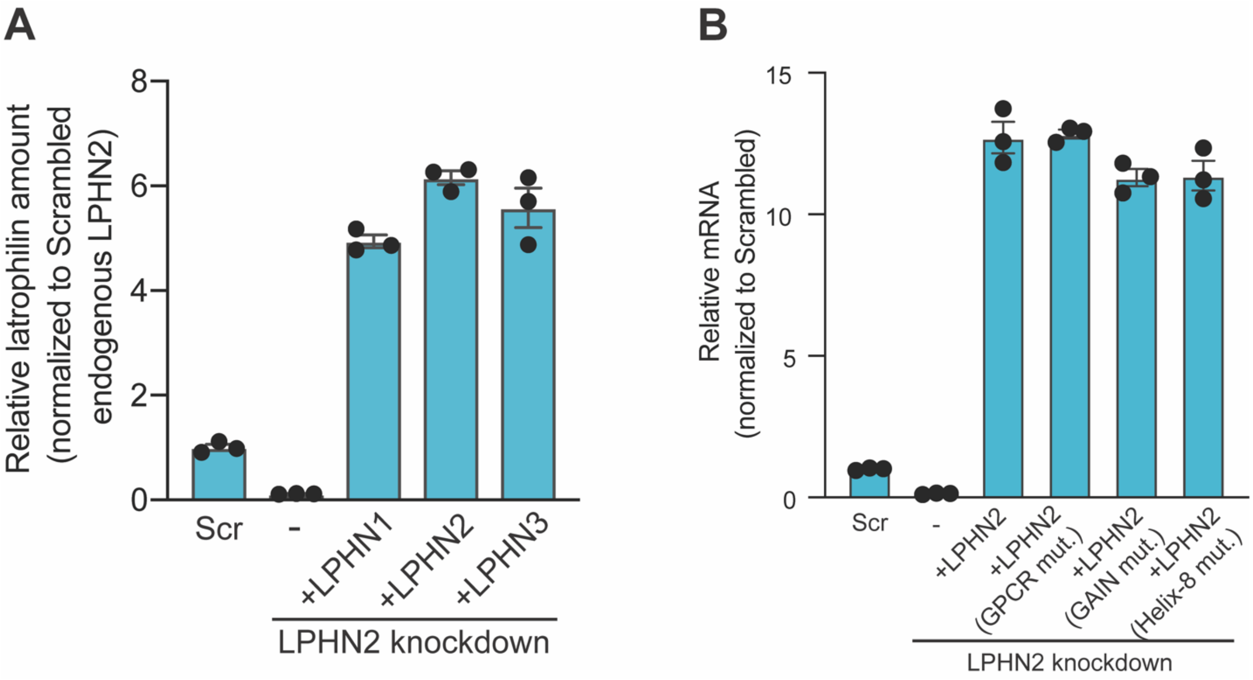

Appendix Figure S8. Validation of knockdown and rescues by Latrophilin mutants

A. mRNA level of LPHN rescue constructs for Fig. 3**K. B**, mRNA level of endogenous LPHN2 and exogenous LPHN2 wild type and its variants for Fig. 3**L**.

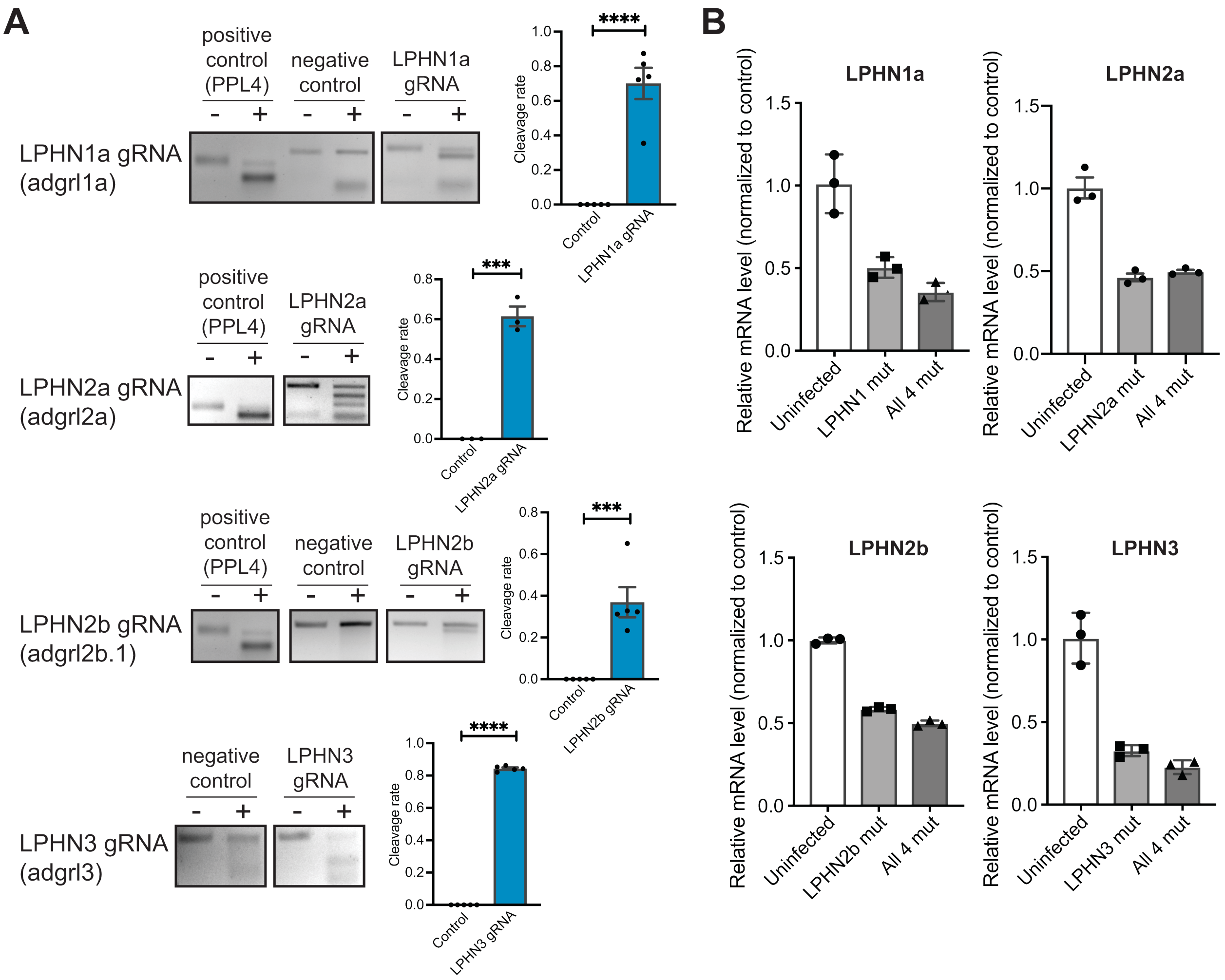

Appendix Figure S9. Validation of latrophilin CRISPR mutant zebrafish

A. T7 endonuclease assay for sgRNA targeting latrophilin isoforms. The positive control is the sample from zebrafish injected with sgRNA for ppl4. Negative control is zebrafish without injection. N=3-5 fish. ***: $p<0.001$, ****: $p<0.0001$; *Student*'s t-test. **B**. RT-PCR for indicated latrophilin isoforms at 48 hours after injection of sgRNA and mRNA encoding Cas9 endonuclease. mRNA degradation of all isoforms due to nonsense mediated decay was observed.

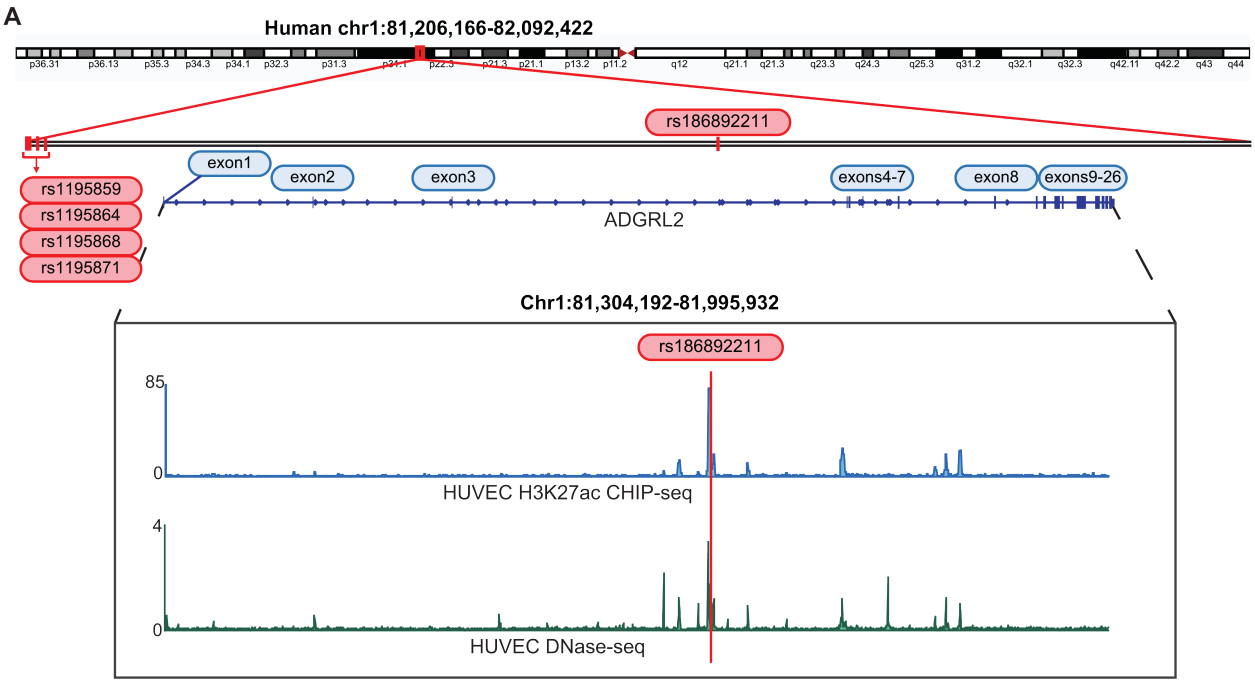

B

Variant (CHR:POS:REF:ALT)	dbSNP	Trait	P-Value	Beta
1:81210578:G:C	rs1195859	Systolic BP	1.413e-7	▲0.0114
1:81212081:C:T	rs1195864	Systolic BP	1.931e-7	▲0.0115
1:81226670:A:G	rs1195868	Systolic BP	2.792e-8	▲0.0110
1:81218249:T:C	rs1195871	Systolic BP	4.821e-8	▲0.0112
1:81705083:C:T	rs186892211	Ischemic stroke	3.13e-10	OR:720.3952

Appendix Figure S10. SNP analysis of human *Adgrl2* gene locus and links to cardiovascular disease

A. Schematic of human *Adgrl2* gene locus on human chromosome 1, H3K27ac CHIP-seq and DNase-seq map of HUVECs obtained from the ENCODE project, and location of SNPs. **B.** Sequence, traits, p-values and odds ratio for each SNP.

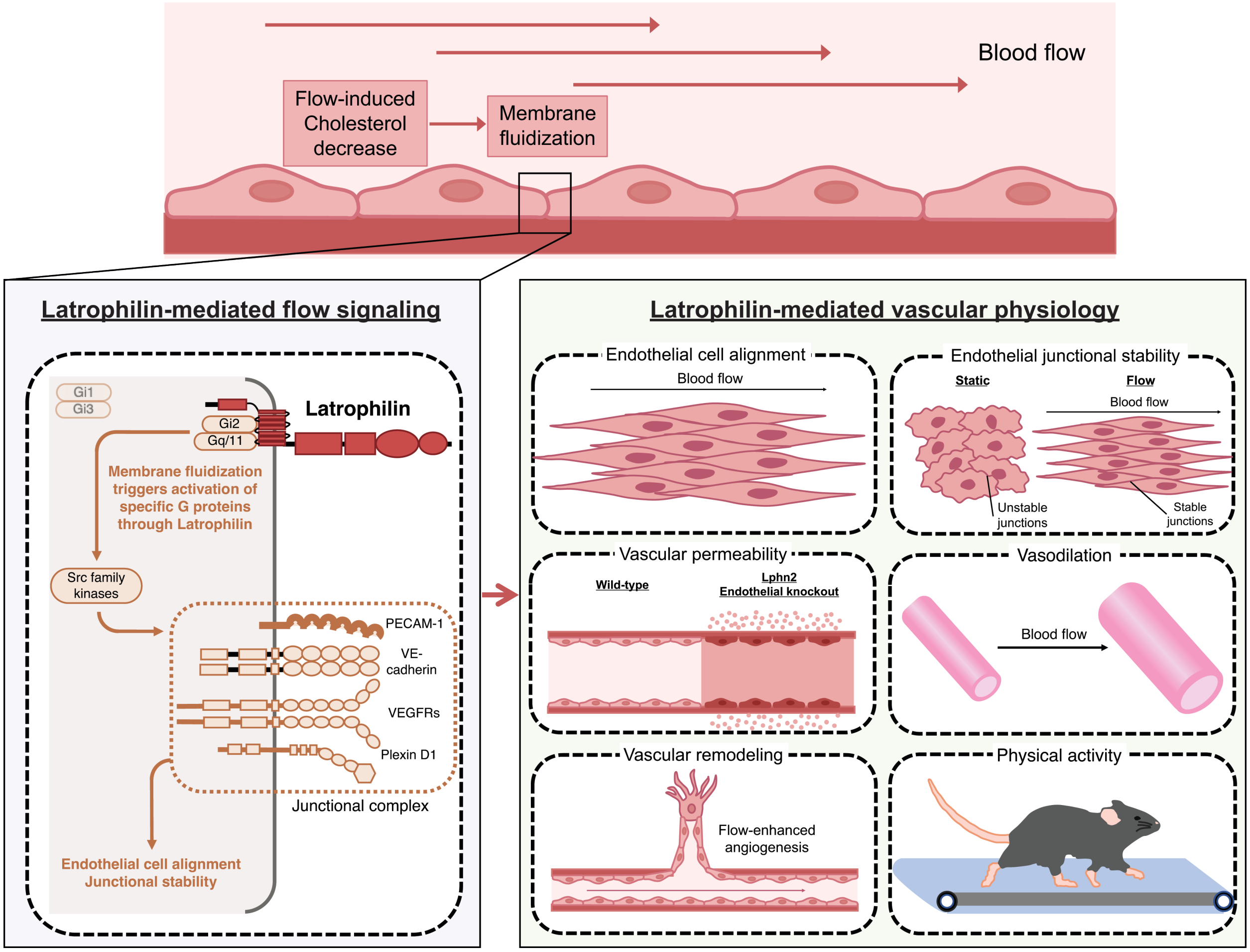

Appendix Figure S11. Latrophilin-mediated endothelial flow signaling pathways and their physiological roles.
