# Supplementary material for: Latrophilin-2 mediates fluid shear stress mechanotransduction at endothelial junctions
Source: EMBO J. 2024 Jun 17;43(15):3175–91. doi: 10.1038/s44318-024-00142-0 (PMC11294477; doi:10.1038/s44318-024-00142-0)
Supplement: Supplementary file 4 — Dataset EV1 [file 44318_2024_142_MOESM4_ESM.docx]

**Dataset EV1. Complete cDNA sequences of G proteins used in this study**

cDNA sequence with silent mutation to make G protein resistant to siRNAs

Gi1 (siRNA-resistant)

atgggctgcacgctgagcgccgaggacaaggcggcggtggagcggagtaagatgatcgaccgcaacctccgtgaggacggcgagaaggcggcgcgcgaggtcaagctgctgctgctcggtgctggtgaatctggtaaaagtacaattgtgaagcagatgaaaattatccatgaagctggttattcagaagaggagtgtaaacaatacaaagcagtggtctacagtaacaccatccagtcaattattgctatcattagggctatggggaggttgaagatagactttggtgactcagcccgggcggatgatgcacgccaactctttgtgctagctggagctgctgaagaaggctttatgactgcagaacttgctggagttataaagagattgtggaaagatagtggtgtacaagcctgtttcaacagatcccgagagtaccagcttaatgattctgcagcatactatttgaatgacttggacagaatagctcaaccaaattacatcccgactcaacaagatgttctcagaactagagtgaaaactacaggaattgttgaaacccattttactttcaaagatcttcattttaaaatgtttgatgtgggaggtcagagatctgagcggaagaagtggattcattgcttcgaaggagtgacggcgatcatcttctgtgtagcactgagtgactacgacctggttctagctgaagatgaagaaatgaaTAGGATGCACGAGTCTATGaaattgtttgacagcatatgtaacaacaagtggtttacagatacatccattatactttttctaaacaagaaggatctctttgaagaaaaaatcaaaaagagccctctcactatatgctatccagaatatgcaggatcaaacacatatgaagaggcagctgcatatattcaatgtcagtttgaagacctcaataaaagaaaggacacaaaggaaatatacacccacttcacatgtgccacagatactaagaatgtgcagtttgtttttgatgctgtaacagatgtcatcataaaaaataatctaaaagattgtggtctcttttaa

Gi2 (siRNA resistant)

atgggctgcaccgtgagcgccgaggacaaggcggcggccgagcgctctaagatgatcgacaagaacctgcgggaggacggagagaaggcggcgcgggaggtgaagttgctgctgttgggtgctggggagtcagggaagagcaccatcgtcaagcagatgaagatcatccacgaggatggctactccgaggaggaatgccggcagtaccgggcggttgtctacagcaacaccatccagtccatcatggccattgtcaaagccatgggcaacctgcagatcgactttgccgacccctccagagcggacgacgccaggcagctatttgcactgtcctgcaccgccgaggagcaaggcgtgctccctgatgacctgtccggcgtcatccggaggctctgggctgaccatggtgtgcaggcctgctttggccgctcaagggaataccagctcaacgactcagctgcctactacctgaacgacctggagcgtattgcacagagtgactacatccccacacagcaagatgtgctacggacccgcgtaaagaccacggggatcgtggagacacacttcaccttcaaggacctacacttcaagatgtttgatgtgggtggtcagcggtctgagcggaagaagtggatccactgctttgagggcgtcacagccatcatcttctgcgtagccttgagcgcctatgacttggtgctagctgaggacgaggagatgaaccgcatgcatgagagcatgaagctattcgatagcatctgcaacaacaagtggttcacagacacgtccatcatcctcttcctcaacaagaaggacctgtttgaggagaagatcacacacagtcccctgaccatctgcttccctgagtacacaggggccaacaaatatgatgaggcagccagctacatccagagtaagtttgaAgaTTTAaaCaaAcgGaaGgacaccaaggagatctacacgcacttcacgtgcgccaccgacaccaagaacgtgcagttcgtgtttgacgccgtcaccgatgtcatcatcaagaacaacctgaaggactgcggcctcttctga

Gi3 (siRNA resistant)

atgggctgcacgttgagcgccgaagacaaggcggcagtggagcgaagcaagatgatcgaccgcaacttacgggaggacggggaaaaagcggccaaagaagtgaagctgctgctactcggtgctggagaatctggtaaaagcaccattgtgaaacagatgaaaatcattcatgaggatggctattcagaggatgaatgtaaacaatataaagtagttgtctacagcaatactatacagtccatcattgcaatcataagagccatgggacggctaaagattgactttggggaagctgccagggcagatgatgcccggcaattatttgttttagctggcagtgctgaagaaggagtcatgactccagaactagcaggagtgattaaacggttatggcgagatggtggggtacaagcttgcttcagcagatccagggaatatcagctcaatgattctgcttcatattatctaaatgatctggatagaatatcccagtctaactacattccaactcagcaagatgttcttcggacgagagtgaagaccacaggcattgtagaaacacatttcaccttcaaagacctatacttcaagatgtttgatgtaggtggccaaagatcagaacgaaaaaagtggattcactgttttgagggagtgacagcaattatcttctgtgtggccctcagtgattatgaccttgttctggctgaggacgaggagatgaaTAGGatgcaCgaGTCTatgaaactgtttgacagcatttgtaataacaaatggtttacagaaacttcaatcattctcttccttaacaagaaagacctttttgaggaaaaaataaagaggagtccgttaactatctgttatccagaatacacaggttccaatacatatgaagaggcagctgcctatattcaatgccagtttgaagatctgaacagaagaaaagataccaaggagatctatactcacttcacctgtgccacagacacgaagaatgtgcagtttgtttttgatgctgttacagatgtcatcattaaaaacaacttaaaggaatgtggactttattga

Gq (siRNA resistant)

atgactctggagtccatcatggcgtgctgcctgagcgaggaggccaaggaagcccggcggatcaacgacgagatcgagcggcagctccgcagggacaagcgggacgcccgccgggagctcaagctgctgctgctcgggacaggagagagtggcaagagtacgtttatcaagcagatgagaatcatccatgggtcaggatactctgatgaagataaaaggggcttcaccaagctggtgtatcagaacatcttcacggccatgcaggccatgatcagagccatggacacactcaagatcccatacaagtatgagcacaataaggctcatgcacaattagttcgagaagttgatgtggagaaggtgtctgcttttgagaatccatatgtagatgcaataaagagtttatggaatgatcctggaatccaggaatgctatgatagacgacgagaatatcaattatctgactctaccaaatactatcttaatgacttggaccgcgtagctgaccctgcctacctgcctacgcaacaagatgtgcttagagttcgagtccccaccacagggatcatcgaatacccctttgacttacaaagtgtcattttcagaatggtcgatgtagggggccaaaggtcagagagaagaaaatggatacactgctttgaaaatgtcacctctatcatgtttctagtagcgcttagtgaatatgatcaagttctcgtggagtcagacaatgagaaccgaatggaggaaagcaaggctctctttagaacaattatcacatacccctggttccagaactcctcggttattctgttcttaaacaagaaagatcttctagaggagaaaatcatgtattcccatctagtcgactacttcccagaatatgatggaccccagagagatgcccaggcagcccgagaattcattctgaaAATGTTTGTTGATTTAAATccagacagtgacaaaattatctactcccacttcacgtgcgccacagacaccgagaatatccgctttgtctttgctgccgtcaaggacaccatcctccagttgaacctgaaggagtacaatctggtctaa
